# Integrating intention-based systems in human-robot interaction: a scoping review of sensors, algorithms, and trust

**DOI:** 10.3389/frobt.2023.1233328

**Published:** 2023-10-09

**Authors:** Yifei Zhang, Thomas Doyle

**Affiliations:** ^1^ Department of Electrical and Computer Engineering, McMaster University, Hamilton, ON, Canada; ^2^ School of Biomedical Engineering, McMaster University, Hamilton, ON, Canada; ^3^ Vector Institute for Artificial Intelligence, Toronto, ON, Canada

**Keywords:** sensor, algorithm, intention (intent), trust, intention-based system

## Abstract

The increasing adoption of robot systems in industrial settings and teaming with humans have led to a growing interest in human-robot interaction (HRI) research. While many robots use sensors to avoid harming humans, they cannot elaborate on human actions or intentions, making them passive reactors rather than interactive collaborators. Intention-based systems can determine human motives and predict future movements, but their closer interaction with humans raises concerns about trust. This scoping review provides an overview of sensors, algorithms, and examines the trust aspect of intention-based systems in HRI scenarios. We searched MEDLINE, Embase, and IEEE Xplore databases to identify studies related to the forementioned topics of intention-based systems in HRI. Results from each study were summarized and categorized according to different intention types, representing various designs. The literature shows a range of sensors and algorithms used to identify intentions, each with their own advantages and disadvantages in different scenarios. However, trust of intention-based systems is not well studied. Although some research in AI and robotics can be applied to intention-based systems, their unique characteristics warrant further study to maximize collaboration performance. This review highlights the need for more research on the trust aspects of intention-based systems to better understand and optimize their role in human-robot interactions, at the same time establishes a foundation for future research in sensor and algorithm designs for intention-based systems.

## 1 Introduction

The advancement of robot systems and machine learning has led to the employment of robots in various industries to restructure labor. According to a report by the International Federation of Robotics (IFR), the adoption of human-robot collaboration is on the rise with an 11% increase in cobot installations compared to 2019 (Xiao et al., 2022). The current robots involved in human-robot interaction scenarios range from taking and serving orders in restaurants to assembling sophisticated parts in factories. However, most interactions with robots require people to approach the robot and initiate the interaction, reflecting their belief in the robot’s ability to complete a successful social encounter (Bartneck et al., 2019). In contrast, humans are both initiators and responders in social interactions, relying on rich sensory input and experience to anticipate the other’s actions. This is where intention-based systems come into play in HRI scenarios.

Intention-based systems are a new class of user-centered assistance systems that recognize the user’s intention and act upon it to take on both active and passive roles in the interaction (Wendemuth et al., 2018). This results in a more natural interaction between the user and the robot as the system synchronizes with the interacting entity during the process. However, the trade-off for this is the need for more sensors to understand the user’s interaction purposes from limited information collected by sensors or provided by the user, such as voice, gesture, eye gaze, etc. Consequently, a multi-modality approach is more common in this system to collect more information from the user, and sensor fusion is employed to generate both the user’s current intention and the prediction of their motive in the long run.

In the field of Human-Robot Interaction, the definition of a “robot” or “intention-based system” has been a subject of debate, particularly with the integration of AI technologies blurring the traditional boundaries. A robot is conventionally considered as an autonomous or semi-autonomous system, capable of perceiving its environment, processing information, and performing actions to achieve specific goals (IEC, 2017). However, the advent of AI has extended this definition to include systems that were not traditionally considered robots. For instance, autonomous vehicles, which have the ability to perceive their environment and operate without human intervention, could be classified as robots within the broader understanding (IEC, 2017). Similarly, exoskeletons, which enable or enhance human capabilities through intelligent design and control, can also be included under this umbrella (IEC, 2017). Moreover, a prime example of this expansive definition is the Da Vinci Surgical System, a robot-assisted platform designed to facilitate complex surgery using a minimally invasive approach in healthcare domain (IEC, 2017). Although it does not operate autonomously, the system enhances the surgeon’s capabilities, enabling more precise movements and greater control, with the need for more communication and teamwork during robotic assisted surgery (RAS) (Randell et al., 2017; Catchpole et al., 2019). This further illustrates how AI-driven systems, even those requiring substantial human operation, can be classified as robots within the context of their intention-based operation. This expanded definition recognizes that as technologies advance, the line distinguishing robots from other systems becomes increasingly ambiguous.

Previous literature has proposed various methods of determining the user’s intention in HRI scenarios by utilizing different sensor data and algorithms. Some of the designs are already employed in working environments such as rehabilitation (Liu et al., 2019a), life-support (Liu et al., 2017), assembly (Mohammadi Amin et al., 2020), driving (Fang et al., 2017), etc. Like machine learning algorithms, intention-based systems are task-oriented in implementation, leading to variability in the choice of sensor combination and algorithm, given the tasks spanning different areas of the industry.

Aside from designing the system, trust is also fundamental in HRI, especially in healthcare. It significantly affects the adoption and optimal use of AI technologies by influencing users’ confidence in the system’s capabilities, reliability, and safety (Choudhury et al., 2022). Trust involves not only belief in the AI’s technical competencies but also understanding its operations, transparency, and risk management (Choudhury et al., 2022). Hence, cultivating trust in HRI is paramount to the successful integration of AI in healthcare and vital for ensuring beneficial interactions between users and AI systems. However, despite the nature of intention-based systems where factors like trust, which influences human interaction with these systems, are pivotal, the exploration of this aspect remains scarce in the existing literature.

### 1.1 Objective

As AI and robotics continue to advance, the use of intention-based systems in working environments is becoming increasingly common (Xiao et al., 2022). However, there is currently no literature providing a comprehensive overview of the design characteristics of intention-based systems. Therefore, a scoping review is needed to gain a better understanding of the field before impactful designs can be made. The aim of this study is to provide a basic understanding of the current state of intention-based systems and assist in future implementations. Specifically, the objectives are to.

1. Provide an overview of the sensors and algorithms used in intention-based systems in the collected experimental research and describe the HRI scenarios in which each system is used.
2. Explore the possible effects of human trust when working with intention-based systems.
3. Identify gaps in literature for future research and establish a foundation for subsequent design.

By compiling these different aspects, this study can help researchers implement more comprehensive and user-friendly intention-based systems in HRI scenarios. The review process will be conducted according to PRISMA (Preferred Reporting Items for Systematic Reviews and Meta-Analyses) guideline (Page et al., 2021) to minimize bias and provide a broad understanding of the current state of the field.

## 2 Methods

This systematic review adheres to the PRISMA guideline throughout the entire process. This guideline outlines a systematic approach to collecting and synthesizing data while having a well-formulated research question. By following this structure, the review aims to provide a comprehensive and unbiased overview of the current status and design characteristics of intention-based systems.

### 2.1 Search strategy

The literature search for this review was conducted between October and November 2022 using three databases: Ovid MEDLINE, Ovid Embase, and IEEE Xplore. The search query used for each database is shown in Table 1. While the syntax of the search string may vary depending on the database, the terms were chosen to capture a similar set of research literature. Both title and abstract, as well as subject headings, were searched and reviewed based on the availability of search methods for each database. The search was limited to English language publications but not restricted by publication date. The aim of the search was to identify as many relevant studies as possible to ensure the comprehensiveness of the review.

**TABLE 1:** Database and respective search strings.

Database	Search string
Ovid MEDLINE, Ovid Embase	1. Exp artificial intelligence/
	2. (Machine intelligence OR Computer intelligence OR Cognitive computing OR Robot* OR Expert system* OR Intelligent system* OR Autonomous agent* OR Artificial* intelligen* OR Machine learning OR Deep learning OR Neural network OR Computational intelligence).ti,ab,sh
	3. (Intent* OR intent* predict* OR move* predict* OR act* predict* OR Prediction algorithm).ti,ab,sh
	4. 1 AND 2 AND 3
IEEE	(“Document Title”:“machine intelligence” OR “Document Title”:“computer intelligence” OR “Document Title”:“cognitive computing” OR “Document Title”:“robot” OR “Document Title”:“expert system” OR “Document Title”:“intelligent system” OR “Document Title”:“autonomous agent” OR “Document Title”:“artificial intelligence” OR “Document Title”:“machine learning” OR “Document Title”:“deep learning” OR “Document Title”:“neural network” OR “Document Title”:“computational intelligence”) AND (“Document Title”:“intent*” OR “Document Title”:“intent* predict*” OR “Document Title”:“move* predict*” OR “Document Title”:“act* predict*” OR “Document Title”:“prediction algorithm”)

### 2.2 Participants

Studies with human aged above or equal to 18 years old are included. The demographics of participants drawn from the surveyed literature present a diverse range. In totality, they comprise of more than 200 individuals spanning various age groups, genders, and physical abilities. The age of participants largely ranged from young adults in their early twenties to individuals in their late sixties, with a few studies focusing on specific age ranges from 21 to 35 years old. In terms of gender, a majority of the subjects were male, though a substantial number of females were also included. The handedness of participants was also considered in some studies in hand gesture recognition, and a few in lower and upper-limb intention recognition included subjects with specific physical conditions, such as amputations. Overall, the participant pool was diverse, providing a broad perspective on the interaction between humans and intention-based systems across different demographic groups.

### 2.3 Intervention

The inclusion criteria for this review were studies that proposed the design of sensors or algorithms for intention-based systems, as well as evaluations of such systems. For the purposes of this review, any system that utilized human intention to provide feedback or judgments was considered an intention-based system, as long as it was implemented in an HRI scenario that directly involved human interaction. Additionally, studies that used Wizard of Oz testing were included in order to provide a comparison, where users believed they were interacting with intention-based systems, but the system was actually controlled by a human.

### 2.4 Inclusion and exclusion criteria

Exclusion criteria was chosen to prune out less relevant literature to this study. Three exclusion criteria (E) and three inclusion criteria (I) are identified as following before screening and assessing the search results from databases.

• E1: Studies that are review articles, dissertations, and conference abstract.
• E2: Studies focusing on financial, cryptocurrency, and brain-computer interface.
• E3: Studies that do not meet the requirement stated in participants, that is, with humans under 18 years of age, or do not meet any inclusion criteria.
• I1: Studies focusing on intention, intention-based system, human-robot interaction.
• I2: Studies that include implementation of sensors or algorithms.
• I3: Studies evaluating effect of intention-based system on team dynamics or human perceptions and attitudes when working with one.

Inclusion criterion I1 required that any study searched had to focus on the topics of intention, intention-based system, or human-robot interaction to be considered for inclusion. Additionally, studies needed to meet at least one of the other inclusion criteria: I2, which included studies that discussed the implementation of sensors or algorithms in intention-based systems, and I3, which included studies that evaluated the effect of intention-based systems on team dynamics or human perceptions and attitudes. However, studies that met any of the exclusion criteria were excluded from the review. E1 excluded all review articles, dissertations, and conference abstracts to ensure that the review was based only on primary sources. E2 excluded studies that focused on irrelevant topics such as financial or cryptocurrency markets, or derivation of human intention in non-HRI scenarios such as brain-computer interfaces. While these topics related to AI methodologies are often incorporated and can be included in the initial search, given that they do not specifically focus on HRI, they will be omitted from the scope of this review. Finally, E3 excluded any study that included a participant of under-aged or did not meet any of the inclusion criteria.

### 2.5 Information extraction

After applying the inclusion and exclusion criteria, full-text articles were reviewed, and data were extracted from the selected references. To ensure the collected data is relevant to the purpose of the review, several crucial aspects were considered, such as the design’s purpose, the sensors and algorithms utilized, the data collection process, the data size for training, testing, and validation, the performance evaluation, demographic information of the participants involved(age, gender, height, weight, right-handed or left-handed), and the method used for identifying intentions. All extracted data can be found in the supplementary files.

Once the data extraction was completed, the identified design characteristics were compiled into a single file and categorized accordingly. A matrix was created to show the statistical outcomes from the references. The designs were then grouped together based on the recognized intention and were analyzed in comparison.

### 2.6 Analysis

**FIGURE 1:**
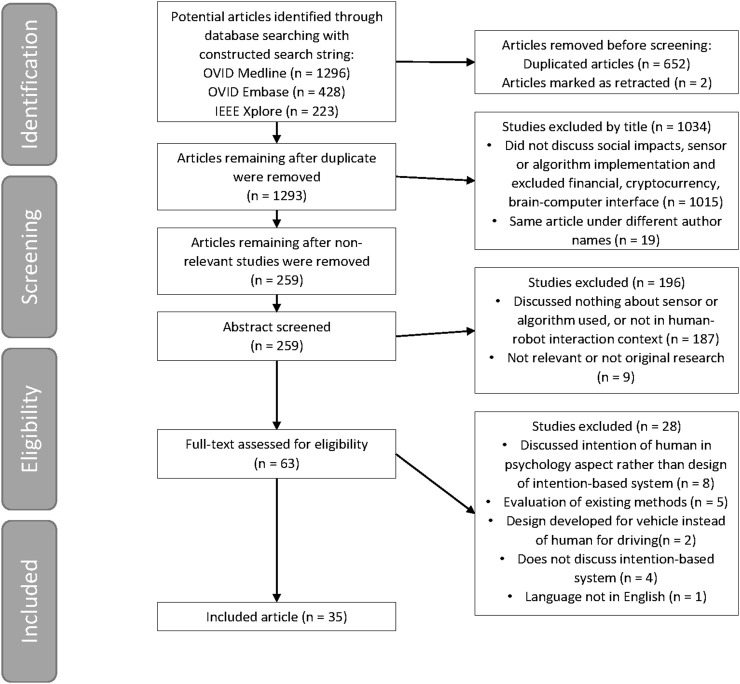
PRISMA (Preferred Reporting Items for Systematic Reviews and Meta-Analyses) flowchart.

The entire process is shown in Figure 1. With the search string in Table 1 applied to the following databases: Ovid MEDLINE, Ovid Embase, and IEEE Xplore. The search identified a total of 1296, 428, and 223 articles, respectively. After removing duplicates and retracted articles, the number of literature reduced to 1293 before screening. All remaining studies were screened by title, and 973 articles (75.25% of deduplicated search) were ineligible for not discussing social impacts, sensor, or algorithm implementation, or is application in financial, cryptocurrency, or brain-computer interface; 19 articles (1.46% of deduplicated search) that are under different author names (problem of naming convention). In the remaining 301 articles, abstract screening excluded 214 articles (71.10%) due to not discuss intention-based system, no sensor or algorithm mentioned, not in HRI context, or not original research. Finally, the remaining 87 articles were screened as full-text, leaving 59 articles (67.82%) as included in this review. The exclusion is more specific compared to the two before: discussed about psychology of intention rather than intention recognition of systems, trials but limited social impact mention, design for recognizing vehicle intention instead of human, etc.

## 3 Results

**FIGURE 2:**
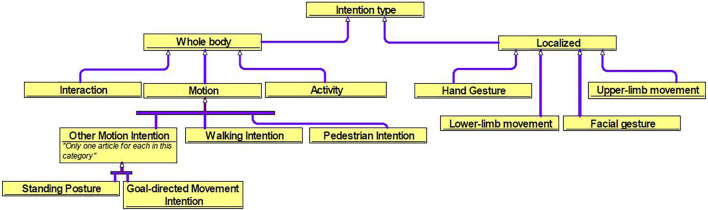
Ontology of intention-based system.

**FIGURE 3:**
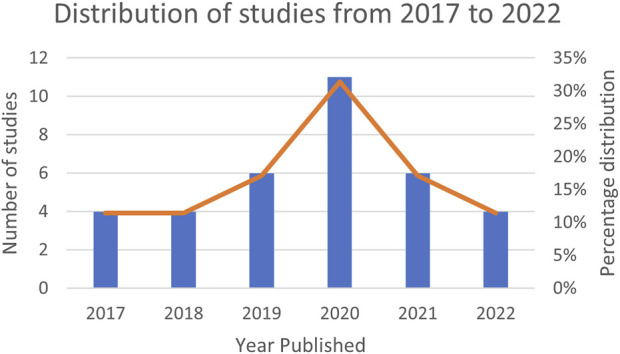
Distribution of studies from 2017 to 2022.

Table 2 presents a summary of the demographic information of the studies included in the review that proposed new designs for sensors and algorithms in intention-based systems. Since the identification of human intention varies depending on the task, the designs proposed in the studies were categorized based on the type of intention, mentioned in Figure 2. It shows the ontology created to better visualize the structure of the literature review. The intention types are separated into whole body and localized body parts, where interaction, motion, and activity are the former, and hand gesture, upper/lower limb movement, facial gesture are the latter. Each would be introduced in the later sections. The use of sensor clusters also varies depending on the specific intention, especially in upper-limb and lower-limb detection, where the placement of electrodes on muscles may differ. Thus, it is difficult to rank the sensors and algorithms used in the designs as being best or worst. Instead, they have their own advantages and disadvantages. Table 3 provides the complete reference to the intention types, and Table 4 for reference to algorithms, which both will be discussed in detail in the later sections. Figure 3 depicts the distribution of included studies from 2017 to 2022, and indicates that interest in intention-based systems has peaked in 2020, although it remains consistent throughout the years.

**TABLE 2:** Summary of study characteristics of sensor and algorithm design literature (n = 35).

Study characteristics	Value, n (%)
Year	
2017	4 (11)
2018	4 (11)
2019	6 (17)
2020	11 (31)
2021	6 (17)
2022	4 (11)
Involved participants	
1–4	6 (17)
5–9	3 (9)
10–15	9 (26)
16–20	2 (6)
21–100	2 (6)
Unspecified	13 (37)
Sensor	
RGB camera	10 (29)
Inertial Measurement Unit (IMU)	6 (17)
Surface electromyography (sEMG)	6 (17)
Depth camera	4 (11)
Force sensor	4 (11)
Myo armband	3 (9)
Custom	5 (14)
Other (mentioned once in article)	11 (31)
Algorithm	
CNN-based	15 (43)
LDA	3 (9)
CNN + ConvLSTM	2 (6)
NN	2 (6)
Other (mentioned once in article)	13 (37)
Data type used	
Image	11
sEMG	10
IMU	3
Force	2
Other (once in article)	9

**TABLE 3:** Literature to intention type.

Intention type	Study
Motion	7 Fang et al. (2017), Liu et al. (2017), Lanini et al. (2018), Ren et al. (2018), Goldhammer et al. (2020), Kumar and Michmizos (2020), Li et al. (2020)
Hand gesture	11 Cote-Allard et al. (2019), Young et al. (2019), Chen et al. (2020a), Chen et al. (2020b), Gardner et al. (2020), Chiu et al. (2021), Feleke et al. (2021), Ding and Zheng (2022), Tsitos et al. (2022)
Lower-limb movement	7 Massalin et al. (2018), Moon et al. (2019), Su et al. (2019), Coker et al. (2021), Viekash et al. (2021), Wang (2021), Wen and Wang (2021)
Upper-limb movement	5 Kilic and Dogan (2017), Liu et al. (2019a), Huang et al. (2020), Kopke et al. (2020), Lu et al. (2020)
Activity	3 Li et al. (2018), Jaouedi et al. (2020), Poulose et al. (2022)
Interaction	1 Mohammadi Amin et al. (2020)
Facial gesture	1 Cha et al. (2019)

**TABLE 4:** Literature to algorithm.

Intention type	Study
CNN-based	15 Fang et al. (2017), Owoyemi and Hashimoto (2017), Su et al. (2019), Chen et al. (2020a), Chen et al. (2020b), Jaouedi et al. (2020), Kumar and Michmizos (2020), Li et al. (2020), Mohammadi Amin et al. (2020), Chiu et al. (2021), Viekash et al. (2021), Wang (2021), Wen and Wang (2021), Ding and Zheng (2022), Poulose et al. (2022)
LDA	3 Lanini et al. (2018), Huang et al. (2020), Kopke et al. (2020)
CNN + ConvLSTM	2 Cha et al. (2019), Zhang et al. (2022)
NN	2 Moon et al. (2019), Coker et al. (2021)
Other (mentioned once in article)	13 Kilic and Dogan (2017), Liu et al. (2017), Li et al. (2018), Massalin et al. (2018), Ren et al. (2018), Liu et al. (2019a), Cote-Allard et al. (2019), Young et al. (2019), Gardner et al. (2020), Goldhammer et al. (2020), Lu et al. (2020), Feleke et al. (2021), Tsitos et al. (2022)

### 3.1 Whole body intention

#### 3.1.1 Motion

The intention of human body movement, including standing posture, gait pattern, and walking, is covered in the section on motion intention. Instead of focusing on specific body parts such as the upper-limb or lower-limb, the studies discussed in this section prioritize whole body movements that are more general. The determination of motion intention can ensure human safety in HRI scenarios by avoiding collisions and increasing efficiency. It differs from activity, as activity focuses on different types of action (e.g., push-up vs. sit-up vs. walking with one algorithm) whereas motion determines the occurrence of one type of action (e.g., only walking). There are a total of 7 articles that falls in the category, a summary of citations can be found in Table 3.

##### 3.1.1.1 Pedestrian intention

One aspect of motion intention is predicting the movement of pedestrians in the context of automated vehicles. Goldhammer et al. (2020) proposed a method called “PolyMLP” which uses artificial neural networks to predict the future movement of pedestrians and cyclists. The method employs a multilayer perceptron (MLP) with sigmoid activation functions and polynomial approximation of time series to recognize the current motion state and future trajectory of vulnerable road users (VRUs). The network is trained using offline learning approach, meaning the model would have no further learning input after training. Since it only requires information regarding VRU’s past position in any coordinate system to make prediction, the sensor choice is widely flexible, and no additional information such as map data is needed. MLP was chosen due to its ability to handle multi-dimensional data input and output and learn complex patterns through several hidden layers. However, MLP requires a large amount of training data and can be prone to overfitting. In this study, the model was trained with video data of pedestrians and cyclists, and the resilient backpropagation (RPROP) algorithm was used to optimize the number and sizes of hidden layers and improve generalization (Riedmiller and Braun, 2002). The trained model classified four motion intentions, including moving, waiting, starting, and stopping, organized into connected states. The method does not require additional information such as map data and is widely flexible in sensor choice. 

Fang et al. (2017) proposed a method to detect a pedestrian’s intention to cross the road or turn in front of the vehicle using stereo camera images as input. The system employs a two-branch multi-stage convolutional neural network (CNN) for recognition of human posture. The CNN is trained on the Microsoft COCO 2016 keypoints challenge dataset for skeleton fitting (Lin et al., 2015), and then a support vector machine (SVM) and random forest (RF) are compared as binary classifiers to determine the motion intention. Both classifiers output a normalized score, with SVM using Platt scaling on radial basis function kernel scores and RF using probability values. The system achieves an identification of crossing intention in under 750 milliseconds when transitioning from other actions such as standing still and bending. The study identifies a problem when encountering pedestrians at a far distance where the skeleton fitting may confuse left and right side body parts. This issue could potentially be minimized with a larger or more specific dataset including these cases.

##### 3.1.1.2 Walking intention

The ability to identify the walking direction of humans is critical in several human-robot interaction scenarios, such as walking support, object manipulation, and exoskeleton control. Liu et al. (2017) proposed a design that can detect the walking direction intention of a human when using a walking support robot (WSR). The design employs a smartphone as a 3-axis accelerometer, along with force sensors embedded in the armrest of the WSR to detect pressure exerted by the human. The accelerometer is placed on the chest of the subject. To classify the intention, SVM is used, which has the advantage of being robust to noise and having global optimization. The training and validation dataset is collected from four participants when using the WSR to walk in eight directions, including forward, back, left, right, left front, left back, right front, and right back. The data is split into 80% for training and 20% for testing. The combined sensors can achieve an accuracy of 89.4% at a data collection window width of 0.1s, and an accuracy of 95.9% at a window width of 0.5s.

Lanini et al. (2018) proposed a model for human-robot collaboration when carrying heavy objects together. The model identifies the motion state of humans and enables the robot to perform synchronous movement. The study used 3D force sensors (Optoforce) and a motion capture system (Optitrack) with 15 markers to collect training data, and only used the force sensor during testing and imaginary work environment. The data collection involved 16 individuals with fair distribution, where one subject always acted as a leader in motion, and the others were followers. The followers were blindfolded and wore earmuffs to prevent visual and acoustic feedback, and the leader was equipped with Bluetooth earphones from which an audible beat was played to minimize disturbances in the data. The feature extraction was performed using single variable classification (SVC) and multivariable classification (MVC) models. SVC was used to investigate the effect of a single threshold approach on features such as force and position, and MVC was not limited in the number of features used. For classification of the four types of identified intentions (Stationary State SS, Walking Forward State WFS, and Walking Backward State WBS), linear discriminant analysis (LDA) classifier was used as it has interpretability on which are the most discriminative features and is fast for training and testing. As a result of supervised learning, SVC performs well on SS, achieving a 96% accuracy, but less satisfactory on WFS with only 79%, while MVC has a 92.3% accuracy for WFS. When the model is implemented on the COMAN robot, it performs well on the starting and stopping of synchronized motion but poorly on acceleration and deceleration, possibly due to misclassification of deceleration as stopping with slow walking speed (0.25 m/s).

Another usage of walking intention detection is in dynamic gait generation for exoskeleton. Ren et al. (2018) designed a on-line dynamic gait generation model to plan real-time gait trajectories in continuous motion process according to user intentions. The exoskeleton used in the study is lightweight lower-limb exoskeleton robot (LLEX), with inertial measurement unit (IMU) in the backpack and angle sensors in the joints. Since the users all have unique stride length, the study adopted a strategy to utilize real-time spatial position planning, then use inverse kinematics to calculate the joint angle trajectory. The walking process is divided into four distinct patterns, start, normal gait, transition, and end, each with different constraint conditions. A two-state state machine is used to distinguish the two-leg support phase and other phases, which the movement intention recognition would focus on. With multi-sensor fusion of the data of IMU and angle sensors and rules developed, the four patterns can be correctly identified, with a 5% difference between generated gait and natural gait collected at the same time of generating. Comparing with existing method proposed by Kagawa et al. (2015), it shows higher accuracy, naturalness, and continuity, among varies stride length tested.

##### 3.1.1.3 Other motion intention

In addition to the previously mentioned motion intentions, there are other categories that require identification.

###### 3.1.1.3.1 Rehabilitation

Kumar and Michmizos (2020) proposed a design to assess motor learning by identifying the intent of initializing a goal-directed movement and the reaction time (RT) of the movement, which can be used in the rehabilitation of sensorimotor impairment. A deep CNN consisting of five layers was trained using 128-channel EEG signals to predict movement intention, and four layers were used for RT classification. Data collection was performed in two separate tasks: the first is active mode, where the subject performs the motion, and the second is passive mode, where motion is performed by the robot with the subject’s arm affixed to the robotic end effector. The training and testing dataset ratio is 4:1, and the mean accuracy achieved for movement intent and RT classification were 87.34%±2.83% and 84.68%±3.68% , respectively. In the future, the proposed model could be used to target specific treatment and provide assistance according to the percentage of voluntary movement, and RT could be used as an indicator of functional motor recovery.

###### 3.1.1.3.2 Standing posture

Li et al. (2020) proposed a design for the recognition of standing posture using a pressure-sensing floor. The floor design includes a pressure buffer layer, a pressure sensor array, and a supporting plate, along with a data collection unit that gathers foot-pressure distribution over the sensor matrix. The foot-pressure distribution is then converted into a grayscale image for further usage. The proposed multi-classifier fusion algorithm includes a CNN similar to lenet-5, a SVM classifier, and a KNN classifier. The latter two were selected after comparing the training results within a group of classifiers that included SVM, KNN, RF, decision tree (DT), Naïve Bayes (NB), and backpropagation (BP) neural network. The trained network can classify between nine standing postures with an average accuracy of 99.96% across testing data. However, this study is limited to static standing postures, and future implementations could focus on identifying dynamically moving subject’s posture.

#### 3.1.2 Activity

This literature review focuses on activity recognition, which involves classifying whole body movements into distinct categories. The studies analyzed in this section utilize image sensors, which are a common method as they convey more comprehensive information (Li and Xie, 2019). Moreover, the sensors do not need to be placed directly on humans which allows activities to proceed without interference. Detecting activities is critical when it comes to collaborating on multiple tasks, as the robot can then identify the specific task the human partner is performing and provide the corresponding assistance. There are a total of 3 articles that falls in the category, a summary of citations can be found in Table 3.

Jaouedi et al. (2020) proposed a novel approach for human activity recognition using a combination of Convolutional Neural Network (CNN) and Recurrent Neural Network (RNN) with a Kalman filter. The CNN model used in this study is a combination of Inception V3 and MobileNet, while the RNN model is used for activity classification. The approach was applied to videos captured using an RGB-D camera and depth camera, and the spatio-temporal features of the human skeleton were extracted for feature presentation. The CAD-60 dataset was used for training and testing the model. The dataset consists of RGB-D video sequences of humans performing activities, recorded using the Microsoft Kinect sensor. The study achieved an accuracy of 95.50% for activity recognition, demonstrating the effectiveness of the proposed approach.

Poulose et al. (2022) proposed a novel approach for Human Activity Recognition (HAR) systems that use a smartphone camera to capture human images and subsequently perform activity recognition. The proposed approach, referred to as the Human Image Threshing (HIT) machine-based HAR system, uses Mask R-CNN for human body detection and ResNet for classification. The HIT machine-based HAR system relies on images captured from a smartphone camera for activity recognition, which has the potential to significantly lower the cost and complexity of HAR systems. The accuracy of the proposed system was evaluated using a dataset of 9 activities, including sitting, standing, walking, dancing, sit-up, running, jumping, push-up, and lying. The model accuracy was reported as 98.53%, with a model loss of 0.20. The precision, recall, and F1 scores were also reported as 98.56%, 98.53%, and 98.54%, respectively. The HIT machine-based HAR system achieved high accuracy in activity recognition, indicating its potential to serve as a cost-effective and efficient solution for HAR systems.

Li et al. (2018) proposes a novel gaze-based intention inference framework for robots. The framework consists of three main components: head pose estimation, eye center localization, and eye model and gaze tracking. By analyzing the gaze data, the system predicts the user’s intention, allowing the robot to provide appropriate assistance or interaction. Existing frameworks mainly focus on establishing the relationship between gaze points and objects, but lack the ability to predict the user’s intentions. The proposed framework aims to address this limitation by enabling the robot to understand the user’s intentions and provide more personalized assistance. The input to the system is gaze data captured by a camera and a fixed monitor of scene image observed by the robot.

#### 3.1.3 Interaction

This literature review underscores the significance of recognizing interaction intention to ensure safety in human-robot interaction (HRI) scenarios. There are instances when humans have no intention of interacting with robots, and it is vital for the robot to identify these moments and halt the collaboration to avoid any potential risks. This category differs from the others, focuses on multimodal approaches, similar to a human-human interaction where multiple sensors (eyes, ears, hands, etc.) are utilized to express intent. There are a total of 1 articles that falls in the category, a summary of citations can be found in Table 3.

Mohammadi Amin et al. (2020) proposed a method to enhance safety by combining visual and tactile perception in human-robot interaction. To achieve this, the study employs a camera system consisting of two Kinect V2 cameras, with RGB and depth cameras. The study utilizes a 3D CNN for human action recognition and 1D CNN for contact recognition. The input for the system includes RGB and depth images captured by the camera system. The dataset for the study consists of 33,050 images divided into five classes of human action recognition and 1,114 samples divided into five classes of contact recognition. The study achieved an accuracy of 99.72% for human action recognition and 93% for contact recognition.

### 3.2 Localized body intentions

#### 3.2.1 Hand gesture

In this literature review, the topic of hand gestures is explored, encompassing both hands and wrist movements. Various studies discussed in this section focus on recognizing different hand gestures and their intended actions. The recognition process involves the use of several sensors and algorithm combinations, such as sEMG, MMG, RGB, and depth sensors. The primary objective of recognizing hand gestures is to enable robots to interpret human actions accurately or follow commands during collaboration, thereby enhancing the efficiency of human-robot interaction (HRI) scenarios. There are a total of 11 articles that falls in the category, a summary of citations can be found in Table 3.

Chen et al. (2020a) proposed a design that utilizes a compact deep neural network called EMGNet for gesture recognition using sEMG data collected by Myo armband. The network has four convolutional layers and a max pooling layer, without a full connection layer as final output. EMGNet has reduced the parameters to 34,311, which is significantly lower than other models, such as CNN_LSTM, LCNN, and ConvNet. Moreover, the accuracy of EMGNet is also higher, with 98.81% on the Myo dataset and 69.62% on the NinaPro DB5 dataset. However, the NinaPro DB5 dataset suffers from low accuracy due to a relatively small amount of data with a large number of gesture categories and similar gestures representing different categories. The Myo dataset has 19 subjects performing 7 gestures, with 2280 samples for each gesture by each person, while the NinaPro DB5 dataset has 10 subjects performing 12 gestures, with 1140 samples for each gesture by each person.

In another study, Chiu et al. (2021) proposed a design for recognizing human intention to open automatic doors by detecting and interpreting hand gestures. The proposed system aims to address privacy concerns and reduce the spread of infection during pandemics by enabling non-contact intention recognition. The authors utilized both thermal and camera sensors to collect data, but only the thermal data was used for actual recognition. The data consists of 6,000 images of RGB and thermal data that were masked into 64 × 48 pixels for “open” and “close” classes. The masked images were then fed into a Mask R-CNN, implemented with the Detectron2 library, to extract human masking. A U-Net structure was subsequently employed to identify the intention of the detected human.

Cote-Allard et al. (2019) presented a novel 3-D printed armband called the 3DC armband for sEMG hand gesture recognition. This armband features a custom SoC that can record 10 sEMG channels in parallel, a 9-axis IMU, a wireless transceiver, a MCU for interfacing the components, and a power management unit (PMU) for low-power consumption. The system is powered by a 100-mAh LiPo battery and employs a Molex connector for connecting with the armband and programming the MCU. For comparative experiment, the performance of the 3DC armband was compared to the widely used Myo armband as an sEMG measurement sensor. The Myo and 3DC armbands were worn simultaneously on the dominant arm of participants, and a total of 8 cycles of 11 hand gestures were collected for testing and training. The ConvNet architecture was then used to classify each gesture. Results showed an accuracy of 89.47% for the 3DC armband and 86.41% for the Myo armband.

Ding and Zheng (2022) proposes a dual-channel VGG-16 CNN for gesture recognition using CCD RGB-IR and depth-grayscale images. The authors collected 30,000 CCD RGB-IR and 30,000 depth-grayscale images using a Kinect depth camera, with 10 actions to recognize in total. They fused the images using three different wavelet fusion techniques (max-min, min-max, and mean−mean), resulting in 30,000 of each fused image. The dataset was split into 15,000 for training and 15,000 for testing. The results showed that the fusion of the min-max type had the highest accuracy of 83.88%, while CCD RGB-IR only had an accuracy of 75.33% and depth-grayscale only had an accuracy of 72.94%. The mean−mean type fusion had an accuracy of 80.95%, which was also relatively high. Overall, the proposed method achieved high accuracy in gesture recognition by combining CCD RGB-IR and depth-grayscale images through wavelet fusion.

In Feleke et al. (2021), a recurrent fuzzy neural network (RFNN) is proposed to map sEMG signals to 3D hand positions without considering joint movements. The aim is to predict human motor intention for robotic applications. The study analyzed the effects of slow and fast hand movements on the accuracy of the RFNN model. Two complex tasks were performed, one involving picking up a bottle from the table and pouring it into a cup, and the other a manipulation task with multiple obstacles, resembling intelligent manufacturing. Each task was performed at both slow and fast speeds, with 9 trials for each scenario, making a total of 36 trials. The accuracy for slow tasks are 83.02%, 81.29% and for fast tasks 85.29%, 82.38%, both in the order of task 1 and task 2. The results showed that RFNN could predict hand positions with high accuracy, regardless of the speed of motion. This approach could be useful for developing human-robot interaction systems.

In Young et al. (2019), a simplified pipeline system for hand gesture recognition is proposed for prosthetic hand users. The system utilizes a Myo armband as the sEMG sensor and a random forest (RF) algorithm for classification. The system was tested on a dataset consisting of five hand gestures: wave in, wave out, spread fingers, fist, and pinch, from the Myo dataset. The results show that the proposed system achieved an accuracy of 94.80%, indicating high performance.

In this study by Gardner et al. (2020), a low-cost multi-modal sensor suite is proposed for shared autonomy grasping, which includes a custom mechanomyography (MMG) sensor, an IMU, and a camera. The system is used to estimate muscle activation, perform object recognition, and enhance intention prediction based on grasping trajectory. The proposed KNN grasp classifier achieved high accuracy for bottle (100%), box (88.88%), and lid (82.46%) grasping tasks. The study aims to overcome limitations of commercially available systems, which often employ indirect mode-switching or limited sequential control strategies. The proposed system allows for simultaneous activation of multiple degrees of freedom (DoF) during grasping. Three different grasp patterns were tested for a box and two different grasp patterns were tested for a bottle and a lid. Each object was tested at 18 different locations, with a 3-s time window provided for the user to reach and grasp the object and 2.5 s to return to a resting position. The results demonstrate the feasibility and potential for shared autonomy grasping using the proposed multi-modal sensor suite.

Tsitos et al. (2022) used a single RGB-D camera to observe human behavior and predict intentions in real-time for robotic actions using logistic regression (LR) algorithm. The aim was to evaluate the feasibility of the proposed approach. The results showed 100% accuracy for both touching and distant objects when compared to human performance in all scenarios. The input was an image, and the dataset consisted of two subjects who performed grasping movements towards two identical objects in two different scenarios. Each participant completed 50 movements towards each object (left or right) for each scenario, making a total of 400 movements. An 85%–15% split was used for training and testing. Further studies are needed to evaluate the proposed approach with a larger sample size and a wider range of scenarios to determine its generalizability and practicality.

From Chen et al. (2020b), a CNN-based algorithm was proposed for stiffness estimation and intention detection using sEMG data collected from the Myo armband. The algorithm consisted of six 2D convolutional layers (Conv2D), a 2D max-pooling layer (MaxPool2D), and three 2D Global Average Pooling layers (GAP2D). The output of the last GAP2D layer was concatenated with the output of the previous layer, and the process was repeated three times. The accuracy of the algorithm was 96% for each type of wrist configurations in several trials.

The ability to identify hand movement intention is prominent for robots on collaborated assembly line in HRI scenario. Zhang et al. (2022) proposed a method to predict human hand motion during an assembly task to improve collaboration flow and efficiency. The design utilizes an RGB camera mounted over the robot, facing downwards at the working area on the table. The study proposed a state-enhanced ConvLSTM network that combines the flexibility and effectiveness of regular ConvLSTM with improved accuracy (Liu et al., 2019b). The experiment involved six sub-tasks, each requiring the installation of one part of a seat. Using an extended Kalman filter (EKF) to track and a separate CNN to recognize the human intended part, together with the ConvLSTM to predict intention, the robot arm can assist the human in assembly with only image data. After training, the recognition accuracy was higher than 99%. Comparing this method with using speech recognition to detect and deliver intended parts, the prediction method saved 36.43 s in completing all sub-tasks. This approach reduces idle time during the process and improves the efficiency and quality of collaboration since longer idle time reflects worse collaboration between human and robot.

Owoyemi and Hashimoto (2017) developed an approach to identify intention by utilizing collections of point clouds. The study used a 3D sensor mapped into 3D occupancy grids and input the processed data into a 3D CNN to recognize arm and hand motion. The evaluation of the model was to recognize subject’s pick and place action from four boxes. Using 119,102 datasets for training and 14,802 separate datasets for offline testing, the model achieved 100% accuracy in identifying the intention of the subject. When compared to LSTM and 1D convolution model variants, the proposed model showed better accuracy with significantly fewer parameters. However, the positioning of the camera used to generate the point cloud can affect the accuracy of the final result. Additionally, generalization can be a problem since only one subject was used in the training and testing datasets, which requires further data collection with different participants and more complex motions to enhance the model’s generalizability.

#### 3.2.2 Upper-limb movement

This literature review delves into the topic of upper-limb movement, which encompasses the entire arm and shoulder, excluding hand gestures and wrist configurations. Various sensors, including sEMG and muscle shape change (MSC), as well as torque and limb position detection by exoskeletons, are utilized to detect upper-limb movements. The primary objective of recognizing upper-limb movement intentions is to enable robots to anticipate the trajectory of human arms and provide assistance in movement, thus reducing the workload for humans in specific muscle areas with effective designs. There are a total of 5 articles that falls in the category, a summary of citations can be found in Table 3.

In Liu et al. (2019a), the focus is on upper-limb rehabilitation using exoskeleton robots, and a study proposing a sensorless control scheme with human intention estimation is discussed. The study aims to address the control problem of upper-limb rehabilitation by utilizing a self-built exoskeleton robot called NTUH-II, which detects shoulder horizontal abduction/adduction (HABD), shoulder flexion/extension (SF), and elbow flexion/extension (EF) joints. The proposed control scheme employs a deep neural network (DNN) for human intention estimation, with the input being the upper limb torque. The accuracy of the scheme was evaluated using root mean square error (RMSE) and normalized RMSE (NRMSE) metrics. The dataset used for training and testing consisted of 28,000 data points and was split into 85%–15% for training and testing, respectively.

Lu et al. (2020) focuses on a study that proposes a novel controller for a Franka Emika robot. The purpose of the study is to enhance the efficiency of human-robot interaction by improving trajectory prediction accuracy. The controller combines variable admittance control and assistant control, and utilizes fuzzy Q-learning and LSTM algorithms for optimization. The input for the controller is human limb dynamics, and the dataset consists of 30 trajectories sampled at 1000 Hz. The trajectory prediction accuracy after model training was less than 1 mm with the actual trajectory. The fuzzy Q-learning algorithm optimizes the damping value of the admittance controller by minimizing the reward function. The LSTM algorithm is utilized to predict the trajectory of the robot based on the human limb dynamics input.

The design and control of an active wrist orthosis that is mobile, powerful, and lightweight is proposed in Kilic and Dogan (2017) as a means to avoid the occurrence and/or for the treatment of repetitive strain injuries. The study utilizes two sEMG sensors at the extensor carpi radialis (ECR) and flexor capi radialis (FCR) muscles, and a force sensor. The control system is based on a fuzzy logic controller. The study aims to reduce the workload of the FCR muscle while maintaining the accuracy of the orthosis. The study recorded the deviation from the intended trajectory for wrist movement and found that it increased from 1.794° to 2.934° on average, but reduced the workload by half for the FCR muscle. The dataset for the study consisted of a healthy person performing an isometric test to record maximum torque and sEMG at the forearm. The orthosis generated three levels of torque according to EMG signals detected at the FCR and ECR muscles.

Kopke et al. (2020) focuses on investigating the effectiveness of pattern recognition of sensor data to identify user intent for various combinations of 1- and 2-degree-of-freedom shoulder tasks. The sensors used in this study include load cells and sEMG electrodes. The dataset consists of different max joint torque lifting or depressing conditions determined by isometric testing. The conditions include 0%, 25%, and 50% of maximum joint torque, with a minimum of three and a maximum of ten trials of each condition completed. Two sets of LDA classifiers were developed for each dataset type, including sEMG, raw load cell data, and a combined dataset, with one set using 0% and ± 25% lifting condition data and the other using 0% and ± 50%. The accuracy of the combined set was found to have a 9.7% error rate.

Huang et al. (2020) presents the development of a novel sensor for the acquisition of muscle shape change (MSC) signals in order to decode multiple classes of limb movement intents. The sensor is custom made using nanogold and is both flexible and stretchable. The study utilized a linear discriminant analysis (LDA) classifier to classify seven classes of targeted upper-limb movements, including hand close, hand open, wrist pronation, wrist supination, wrist extension, wrist flexion, and rest state. The dataset was collected with a video prompt for each movement, followed by a rest session, with each prompt lasting 5 s. The accuracy achieved was 96.06% ± 1.84%, demonstrating the potential of using MSC signals for multi-class limb movement intent recognition.

#### 3.2.3 Lower-limb movement

This literature review explores the topic of lower-limb movement, which pertains to leg motions. As walking intention is considered a part of the overall body movement, this section places greater emphasis on detecting signals from the lower body to determine related activities and partial movement intentions of the thigh, knee, and other such areas. The primary objective of recognizing lower-limb movement intentions is to enable robots to predict and assist humans in various movements, including walking, standing, and stair ascending/descending, among others. There are a total of 7 articles that falls in the category, a summary of citations can be found in Table 3.

In Moon et al. (2019), the development of a single leg knee joint assistance robot with motion intention detection using a sliding variable resistor to measure length between the knee center of rotation and the ankle (LBKA) was investigated. The aim of the study was to enhance the control of exoskeletons by incorporating the detection of motion intention. The algorithm used in the study was a neural network with 15 hidden layers and one input/output layer. The dataset used in the study consisted of three motions: stairs ascending, stairs descending, and walking. 40 training datasets were used for each motion, and 200 training datasets were used for exception state training. The performance of the algorithm was evaluated based on the ROC curve. The results showed that the algorithm had good performance, indicating that it is a promising approach for motion intention detection in exoskeletons.

Su et al. (2019) proposed a novel method for training an intent recognition system that provides natural transitions between level walk, stair ascent/descent, and ramp ascent/descent. The study utilizes three IMUs (thigh, shank, and ankle of the healthy leg) and a CNN algorithm for motion intent recognition. The input to the system is lower limb IMU data, and the dataset consists of 13 classes of motion intent. The able-bodied individuals performed ten trials each, with at least five steps, while the amputees performed ten locomotion modes, including level ground, stairs, and ramp, and any transition between them. The dataset comprises 1300 samples from able-bodied individuals and 130 from amputees. The accuracy of the system is 94.15% for able-bodied individuals and 89.23% for amputees.

In Wen and Wang (2021), a lower-limb motion intention recognition algorithm is proposed that utilizes multimodal long-term and short-term spatiotemporal feature fusion for accurate recognition. The input used for the algorithm is sEMG data, and the proposed algorithm consists of a 3D CNN for extracting short term spatiotemporal features in segments, Le Net and shape context to extract features of the target motion trajectory, and an LSTM network for time-series modeling of the extracted features. The purpose of the study is to develop a robust motion intention recognition system that can accurately interpret human motion in real-time scenarios. The accuracy achieved by the proposed algorithm is 90%, indicating that the fusion of long-term and short-term spatiotemporal features has significantly improved the recognition performance. However, the study does not provide any information regarding the dataset used for testing the proposed algorithm.

The study of Massalin et al. (2018) proposes a user-independent intent recognition framework using depth sensing for five activities: standing, walking, running, stair ascent, and stair descent. The objective of the study is to develop and validate a framework that can accurately recognize user intention without relying on user-specific data. The sensor framework consists of a depth camera on the shank and an action camera for class labeling, with support vector machine (SVM) as the algorithm for classification. The dataset includes 5 activities with 20 trials per subject, resulting in a total of 402403 depth images. The study concludes that the proposed framework can accurately recognize user intention in real-time, which could have potential applications in various fields such as sports training, gait analysis, and rehabilitation. The framework’s user-independent nature makes it particularly useful in scenarios where user-specific data cannot be obtained, such as in public spaces or medical facilities. The accuracy of the proposed framework is reported to be 94.5%, which is achieved using 8 subjects’ data for training and 4 subjects for testing.

Wang (2021) proposes the use of a convolutional neural network (CNN) model to reconstruct the motion pattern of a lower limb prosthesis. The input to the CNN model is the data collected from the IMU sensor attached to the prosthesis. The dataset used in this study includes four different motion patterns: heel strike, support, swing, and tippy toes touchdown. The CNN model used in this study includes seven layers, which are used to extract the features from the input data and classify the motion pattern. The accuracy of the system is measured using a recognition rate, which is found to be 98.2%. The use of a single sensor and the high accuracy of the system make it a practical and convenient solution for motion pattern recognition in lower limb prostheses.

Coker et al. (2021) presents a method for predicting knee flexion angle using surface electromyography (sEMG) signals from thigh muscles and knee joint angle data. The purpose of this research is to develop a framework for predicting human intent for control purposes in exoskeleton technology. Twelve sEMG electrodes and a 10-camera Vicon motion capture system were used to collect data from ten subjects during walking trials. A nonlinear input-output time series neural network trained using Bayesian regularization was used to predict the knee’s flexion angle at 50, 100, 150, and 200 ms into the future. The neural network consisted of a single hidden layer of ten nodes with a feedback delay set to two. The accuracy of the predictions was evaluated using RMSE, which was found to be 0.68 for 50 ms, 2.04 for 100 ms, 3.38 for 150 ms, and 4.61 for 200 ms. The results showed good accuracy in predicting the knee joint angle up to 100 ms in advance, which is promising for real-time control of exoskeletons. However, the accuracy decreased for longer prediction horizons, which may be due to the complexity of the underlying muscle activation patterns. The dataset included ten subjects with no history of chronic pain in the spine or lower extremities, which suggests that the results may not be generalizable to individuals with injuries or pathologies.

Viekash et al. (2021) presents a new approach for controlling and actuating a continuous passive motion (CPM) machine using a deep learning-based control strategy that integrates CNNs. The sensor inputs include sEMG and thigh IMU data, which are used to train three 1D-CNN models. Each 1D CNN algorithm is employed to analyze the sensor data, and 40 trials are conducted for each motion (forward, backward, and rest) during the training phase. The training and testing datasets are split at an 80%–20% ratio. The accuracy of the proposed approach is reported to be 97.40%, indicating good performance.

#### 3.2.4 Facial gesture

This literature review highlights the importance of facial gestures in controlling augmented reality/virtual reality (AR/VR) systems. These gestures can potentially be utilized to collaborate with robots and enhance the efficiency of such collaborations.

In Cha et al. (2019), an infrared (IR) camera and laser diode were used to capture skin deformation data as input for a spatial-temporal autoencoder (STAE) that recognizes facial gestures for hands-free user interaction (UI) with an augmented reality (AR) headset. The use of skin deformation as input for gesture recognition is a novel approach to hands-free UI for AR headsets. The STAE consists of two 3D convolutional neural networks (CNN), two convolutional long-short-term memory (ConvLSTM) networks, and one 3D CNN. The goal was to achieve high accuracy in recognizing user intentions based on facial gestures. The results showed an accuracy of 95.4% on 10 subjects during data collection. Future work could investigate the use of this approach in real-world AR applications, as well as explore the potential for combining facial gesture recognition with other types of input, such as voice or gaze, to further enhance hands-free UI for AR.

## 4 Discussion

As robotics become more integrated into our working and living environments, ensuring the safety and efficiency of human-robot interaction has become increasingly important. Intention-based systems have emerged as a promising approach to achieve this, as they allow robots to anticipate and respond to human movements and intentions. This literature review provides an overview of the current methods used in implementing intention-based systems, with a specific focus on the sensors and algorithms used in the process. Various studies have proposed designs for different task environments to react to different determined intentions. However, due to the current limitations of sensors and algorithms, it is premature to assume that one combination of sensors and algorithms is the best choice for all tasks. Therefore, further research is needed to determine the most effective sensor and algorithm combinations for specific human-robot interaction scenarios.

When analyzing the data extracted from the literature and presented in Table 2, it becomes clear that the most popular sensor used in designs is the RGB camera (Fang et al., 2017; Li et al., 2018; Gardner et al., 2020; Jaouedi et al., 2020; Mohammadi Amin et al., 2020; Chiu et al., 2021; Ding and Zheng, 2022; Poulose et al., 2022; Tsitos et al., 2022; Zhang et al., 2022). This is likely due to the widespread use of image recognition applications in recent years, as well as the relative affordability and accessibility of RGB cameras in work environments. RGB cameras have a distinct advantage in intention recognition due to their ability to capture detailed color information. This allows AI systems to accurately perceive and understand human actions in real-world scenarios, enhancing the robot’s ability to infer human intent. By capturing rich visual data, RGB cameras enable machine learning models to interpret nuanced human behaviors and gestures, improving the robot’s ability to anticipate human actions and interact more naturally and efficiently. However, they also have notable disadvantages. RGB cameras may struggle with recognizing intentions in low light conditions or when the subject is at a distance. Additionally, they can be affected by occlusion, where objects in the foreground block those in the background. Lastly, there are significant privacy concerns associated with using RGB cameras for intention recognition, as they can capture identifiable and sensitive visual data.

The second most commonly used sensors are IMUs and sEMG sensors. IMUs are often found in exoskeleton designs and commercialized armbands, such as the Myo armband (Ren et al., 2018; Cote-Allard et al., 2019; Su et al., 2019; Gardner et al., 2020; Viekash et al., 2021; Wang, 2021), while sEMG sensors are mainly used to measure upper-limb movements with armbands (Cote-Allard et al., 2019; Young et al., 2019; Chen et al., 2020a; Chen et al., 2020b) and lower-limb movements with sEMG electrodes (Kilic and Dogan, 2017; Kopke et al., 2020; Coker et al., 2021; Feleke et al., 2021; Viekash et al., 2021; Wen and Wang, 2021). IMUs, which measure body acceleration and angular rate, offer the advantage of being unobtrusive, portable, and relatively easy to use, making them ideal for real-time, dynamic motion tracking. However, their accuracy may be affected by sensor drift over time, and they may not capture subtle movements or muscular activities that do not result in noticeable motion. On the other hand, sEMG sensors, which record muscle activation, offer high temporal resolution and can detect subtle muscle contractions that might not result in visible motion, potentially improving the detection of intended movements. They can provide detailed information about the degree of muscle activation, which can be useful in assessing user intent in tasks that require fine motor control. However, sEMG signals can be sensitive to variations in sensor placement, skin condition, and muscle fatigue, which can affect their reliability. Also, the setup of sEMG sensors can be more obtrusive and uncomfortable for the user, which might limit their use in certain scenarios.

In contrast, force sensors and depth cameras are less popular. Force sensors have limitations regarding the area and tasks that require contact, which may account for their relatively low usage in designs. Additionally, depth cameras are often used in conjunction with RGB cameras rather than being employed as a standalone sensor in designs. They may also struggle with distant subjects. Moreover, they can have difficulties with transparent or reflective surfaces, and their depth accuracy decreases as the distance from the camera increases.

When compared to other algorithms, those based on CNN have been used most frequently in intention recognition research. This is likely due to the popularity of RGB cameras, which capture visual input that can be processed by CNNs to identify patterns or features indicative of specific motions or actions. They excel in feature extraction from images, which makes them ideal for interpreting complex patterns and details in RGB images. The result can be a more accurate, real-time prediction of intentions based on visual cues, gestures, and behaviors captured by the RGB camera and analyzed by the CNN. Furthermore, CNNs are well-suited to handling complex and high-dimensional datasets, which are often generated when multiple sensors are used simultaneously in intention recognition.

The majority of studies are focused on motion intention-based system. There appears to be an even spread in more specific directions such as walking intention, pedestrian intention, and hand motion. The most common sensor and algorithm used to determine motion intents are cameras and CNN. Since motion intention is about whole body movement, sEMG and IMU are not as descriptive as camera sensor with the same effort, which builds up to a lot of CNN usage.

Several studies also focus on recognizing hand gestures, which are often used to issue commands to robots or signal collaboration intent. In these studies, sEMG and RGB sensors are used in relatively equal numbers. This is because sEMG sensors, when used on armbands, can accurately predict hand motion, while cameras can capture detailed visual information about hand gestures. Additionally, there has been an increasing usage of sEMG in upper-limb and lower-limb intention determination, making it an ideal choice when only partial intention needs to be determined. This occurs when only a small group of related muscles are used to complete an intended action. While CNN-based algorithms remain the majority in intention recognition research, various other algorithms and classifiers are also used, including RFNN, RF, and KNN. This is because some designs employ sEMG sensors, which can classify hand gestures without requiring complex data input. As a result, CNNs may not always be necessary for these specific applications, leading researchers to explore alternative algorithms and classifiers.

While motion-based systems have been widely explored in intention recognition research, there are other domains that have received less attention and present opportunities for further study. For example, interaction and facial gestures are relatively unexplored areas that could benefit from more research.

Interaction can be studied in both social robot and industrial robot contexts, although the literature review focused primarily on the latter, with the expanded definition described in the introduction. One included study (Mohammadi Amin et al., 2020) proposed a design for ensuring safety during interactions with robots using visual and tactile perception, which initiated research on combining tactile cues with intention-based systems. In addition, as collaboration between humans and robots is most efficient when communication is bidirectional, it is also important to explore methods for recognizing the intentions of robots, as this will enable more effective collaboration.

Facial gesture can be explored further for integration with other intention recognition in working environment. For example, a robot could be programmed to recognize specific facial expressions, such as frustration or confusion, and use this information to adjust its actions accordingly. This could be used as an additional factor for robot reaction, improving the robot’s ability to support human operators in various tasks. Additionally, facial gesture recognition could be used in assistive robots, allowing users to control the robot’s behavior using facial expressions and gestures, leading to more intuitive communication between the user and the robot.

The impact of intention-based systems on trust in HRI scenario has not been extensively studied. None of the articles related to sensor and algorithms included in this review have considered the effect of trust on participants. While there is a lack of specific research on the effects of these systems on trust, general research on robots and AI in healthcare domain can provide some insight. The studies conducted by Choudhury et al. (2022), Esmaeilzadeh et al. (2021), Asan et al. (2020) underscore the multifaceted importance of trust in the implementation of AI within healthcare scenarios, as it emerges as a pivotal factor influencing the acceptance and effective use of these technologies, as well as Torrent-Sellens et al. (2021), which highlights the factors affecting trust in RAS.

Choudhury et al.’s study (Choudhury et al., 2022) offers a targeted perspective by examining clinicians’ trust in AI systems and how this influences their willingness to adopt such technologies. Notably, trust appears to serve as a mediator between perceived risk and expectancy in the decision to use the AI tool. This study underscores the importance of striking a balance between trust and over-reliance, suggesting that an informed and rational level of trust leads to an optimal utilization of AI, whereas blind trust can lead to overdependence and potential misuse.

Esmaeilzadeh et al. (2021) broaden the perspective by focusing on patients’ perceptions and how they interact with trust. Their study identifies an array of factors - from privacy concerns to communication barriers - that could influence patients’ trust in AI and subsequently their intention to use AI in their healthcare. The study particularly emphasizes the importance of physician involvement in healthcare delivery involving AI tools, suggesting a co-existence model where AI augments rather than replaces human care providers.

Another study by Asan et al. (2020) discussed a similar aspect. The study reflected that trust varies between patients and clinicians. With the rise of patient-centered care, understanding the role of AI in patient-clinician decision-making is essential. Concerning medical responsibility, clinicians could face accountability if AI recommendations deviate from standard care, leading to negative outcomes. Thus, it is crucial to find a balance of trust between human judgment and AI recommendations, considering the evolving nature of AI and individual human factors.

The study by Torrent-Sellens et al. (2021) examines factors affecting trust in robot-assisted surgery in Europe. Trust initially increases with more experience with robots but declines as this experience grows, suggesting a nuanced relationship. Sociodemographic factors play a pivotal role; men, those aged 40–54, and higher-educated individuals show pronounced trust based on their experience. Access to detailed, accurate information about procedures significantly impacts trust. The study calls for public policy should address the fluctuating trust by funding research on regulatory, ethical, and legal aspects and emphasizing clinical efficacy, as current design and model lacks attention on the importance of trust during RAS.

While the studies mentioned above shed some light on trust of human with robots and AI in healthcare, they do not specifically address the potential impact of intention-based systems in human-robot interaction scenarios. Intention-based systems are designed to be more “intelligent” and responsive to human intention and behavior, which may lead to different characteristics and perceptions of the robot by the human team members. As robots become more integrated into various aspects of human society, it is crucial to examine the effect of intention-based systems on trust and cooperation in different settings, such as in industrial or medical contexts. Further research on intention-based systems can provide insight into how to design and implement such systems to gain adequate trustand cooperation between humans and robots in various contexts.

## 5 Conclusion

This literature review follows the PRISMA guidelines and examines the sensors and algorithms used in intention-based systems, as well as their potential impact on trust and team dynamics. The studies included in this review propose various designs for intention-based systems based on the given task environment, with the choice of sensors and algorithms being dependent on the task at hand. RGB cameras and CNN-based algorithms are the most commonly used sensors and algorithms, respectively. In contrast, sEMG measurements in electrodes and armbands are more commonly used for determining partial body intention, such as for upper-limb and lower-limb.

Despite the advancements in intention-based systems, there are still several areas where further research is needed. For instance, interaction intention can be further explored to improve bidirectional communication and increase the efficiency of collaboration. Facial gesture recognition could be integrated with other intention recognition methods to create a more intuitive interaction environment. Additionally, the effect of intention-based systems on trust and team dynamics in HRI scenarios has not been well studied. Finally, there is a need to investigate the impact of anthropomorphism on the perception of robots in moral interactions.

This literature review provides a foundation for future research and development of intention-based systems, as well as analysis of their social impact factors. By exploring the gaps in the existing literature, future research can help improve the effectiveness and safety of human-robot interactions in various industries.

## Data Availability

The original contributions presented in the study are included in the article/supplementary material, further inquiries can be directed to the corresponding author.
